# Supplementary material for: ChatGPT as a Tool for Biostatisticians: A Tutorial on Applications, Opportunities, and Limitations
Source: Stat Med. 2025 Oct 23;44(23-24):e70263. doi: 10.1002/sim.70263 (PMC12548020; doi:10.1002/sim.70263)
Supplement: Supplementary file 1 — Data S1. Supporting Information. [file SIM-44-0-s001.zip › Supplementary Materials Additional.zip!/3.7_Translation_Between_Multiple_Statistical_Programming_Languages/3.7.2_SPSS_to_R/3.7.2_Translation_Chat4.pdf]

R 4

Clear memory

I can't clear memory directly, but you can manage or clear memory by going to **Settings > Personalization > Memory**. From there, you can view what I remember and delete anything you'd like.

Let me know if you want help navigating to that!

Take the role of an expert biostatistician for this chat

Got it! I'll take on the role of an expert biostatistician for this chat. Feel free to throw any questions my way—whether it's study design, statistical modeling, survival analysis, clinical trial methods, or interpreting tricky results. What are we working on today?

Thanks for the context! You're running a **forward stepwise logistic regression using likelihood ratio tests** in SPSS, with V2 as the binary outcome and predictors V3 through V32.

Let's replicate this in **R**, using `glm()` for logistic regression and `step()` for stepwise selection. The `step()` function performs stepwise model selection by AIC, which is the closest approximation to SPSS's likelihood ratio method when working in R (SPSS uses likelihood ratio chi-square tests, while R's `step()` uses AIC by default).

🔁 SPSS to R Translation

Here's the full R translation of your SPSS command:

```r
# Convert V2 to binary (TRUE = Malignant)
data$V2 <- as.factor(data$V2)

# Fit the null model (intercept only)
null_model <- glm(V2 ~ 1, data = data, family = binomial)

# Full model with predictors V3 to V32
full_model <- glm(V2 ~ ., data = data[, -1], family = binomial)

# Perform forward stepwise selection using AIC (like SPSS FSTEP(LR))
stepwise_model <- step(null_model,
                       scope = list(lower = null_model, upper = full_model),
                       direction = "forward",
                       trace = TRUE)
```

Notes:

- We drop `V1` (`data[, -1]`) since it's an ID column in the UCI dataset.

- `step()` uses AIC, which penalizes model complexity. It's not an exact match for SPSS's PIN(0.05) and POUT(0.10), but similar in spirit.
- If you want a selection based on p-values like SPSS, you'd need to use custom code or a package like `MASS::stepAIC` (which still uses AIC) or `olsrr` (for p-value-based selection, more akin to SPSS).

Would you like me to show you how to replicate SPSS-style entry/removal p-value thresholds in R (e.g., PIN=0.05, POUT=0.10) using a different package or custom logic?

Yes

Perfect. To replicate **SPSS-style forward stepwise logistic regression** in **R** using **p-value thresholds** (e.g., `PIN = 0.05`, `POUT = 0.10`), you'll need a package that supports this functionality because base R's `step()` uses AIC, not p-values.

✅ Recommended package: `olsrr`

While `olsrr` is primarily for linear regression, it provides a good framework for **p-value-based stepwise selection**. But for **logistic regression**, we'll take a custom approach using `glm()` and likelihood ratio tests.

Here's how to manually implement **forward stepwise logistic regression** using **likelihood ratio tests** in R, closely mimicking what SPSS does:

📦 Step 1: Setup

```r
# Load necessary packages
library(dplyr)

# Ensure outcome is binary factor
data$V2 <- as.factor(data$V2)
```

🔁 Step 2: Forward Stepwise Logistic Regression by LRT (Custom Function)

```r
forward_stepwise_lrt <- function(data, response, predictors, pin = 0.05, pout = 0.10) {
  selected_vars <- c()
  remaining_vars <- predictors

  # Start with null model
  model <- glm(as.formula(paste(response, "~ 1")), data = data, family = binomial)

  repeat {
    # Try adding each variable one at a time
    pvals <- sapply(remaining_vars, function(var) {
      temp_model <- glm(as.formula(paste(response, "~", paste(c(selected_vars, var), collapse = "+"
                        data = data, family = binomial)
      # Likelihood ratio test
      pval <- anova(model, temp_model, test = "LRT")$`Pr(>Chi)`[2]
```

```
      return(pval)
    })

    min_p <- min(pvals, na.rm = TRUE)
    min_var <- remaining_vars[which.min(pvals)]

    # If the smallest p-value is below PIN, add the variable
    if (!is.na(min_p) && min_p < pin) {
      selected_vars <- c(selected_vars, min_var)
      remaining_vars <- setdiff(remaining_vars, min_var)
      # Update the current model
      model <- glm(as.formula(paste(response, "~", paste(selected_vars, collapse = "+"))),
                   data = data, family = binomial)
    } else {
      break
    }
  }

  return(summary(model))
}
```

🧪 Step 3: Apply to Your Data

Assuming your data frame is called `data`, your outcome is `V2`, and your predictors are `V3` to `V32`:

```
r

predictors <- paste0("V", 3:32)
forward_stepwise_lrt(data, response = "V2", predictors = predictors, pin = 0.05, pout = 0.10)
```

📝 What this does:

- Starts with no predictors.
- Adds one variable at a time using the **likelihood ratio test**, only if the p-value is **< 0.05 (PIN)**.
- Stops when no remaining variable meets the threshold.
- Mimics the **SPSS FSTEP(LR)** method with `PIN=0.05`.

Would you like to also include backward elimination (POUT = 0.10) after each forward step for full SPSS-style behavior?

Yes

Awesome — you're after a **full SPSS-style forward stepwise logistic regression**, meaning:

- **Forward selection**: Add variables if *p* from likelihood ratio test < **PIN = 0.05**
- **Backward elimination**: After each addition, remove any previously included variables if *p* > **POUT = 0.10**

Here's a full custom R function to replicate this process closely:

🧠 Full SPSS-style Forward Stepwise Logistic Regression in R

```r
r

forward_stepwise_sp <- function(data, response, predictors, pin = 0.05, pout = 0.10) {
  selected_vars <- c()
  remaining_vars <- predictors
  model_formula <- as.formula(paste(response, "~ 1"))
  model <- glm(model_formula, data = data, family = binomial)

  repeat {
    # --- FORWARD STEP ---
    forward_pvals <- sapply(remaining_vars, function(var) {
      temp_formula <- as.formula(paste(response, "~", paste(c(selected_vars, var), collapse = "+"))
      temp_model <- glm(temp_formula, data = data, family = binomial)
      pval <- anova(model, temp_model, test = "LRT")$`Pr(>Chi)`[2]
      return(pval)
    })

    min_p <- min(forward_pvals, na.rm = TRUE)
    min_var <- remaining_vars[which.min(forward_pvals)]

    if (!is.na(min_p) && min_p < pin) {
      selected_vars <- c(selected_vars, min_var)
      remaining_vars <- setdiff(remaining_vars, min_var)
      model <- glm(as.formula(paste(response, "~", paste(selected_vars, collapse = "+"))),
                   data = data, family = binomial)
    } else {
      break  # No variables meet the PIN threshold
    }

    # --- BACKWARD STEP ---
    repeat {
      if (length(selected_vars) < 2) break  # Need at least 2 to compare

      pvals_back <- sapply(selected_vars, function(var) {
        reduced_vars <- setdiff(selected_vars, var)
        reduced_model <- glm(as.formula(paste(response, "~", paste(reduced_vars, collapse = "+"))),
                             data = data, family = binomial)
        pval <- anova(reduced_model, model, test = "LRT")$`Pr(>Chi)`[2]
        return(pval)
      })

      max_p <- max(pvals_back, na.rm = TRUE)
      if (!is.na(max_p) && max_p > pout) {
        remove_var <- selected_vars[which.max(pvals_back)]
        selected_vars <- setdiff(selected_vars, remove_var)
        model <- glm(as.formula(paste(response, "~", paste(selected_vars, collapse = "+"))),
                     data = data, family = binomial)
      } else {
        break  # No variables exceed POUT threshold
      }
    }
  }

  return(summary(model))
}
```

🧪 Run It on Your Dataset:

```r
r

# Make sure outcome is a factor
data$V2 <- as.factor(data$V2)

# Define predictors (e.g., V3 to V32)
predictors <- paste0("V", 3:32)

# Run SPSS-style stepwise logistic regression
final_model <- forward_stepwise_sp(data, response = "V2", predictors = predictors)
```

🧾 Output:

You'll get a `summary()` of the final logistic regression model after all steps — just like SPSS would give at the end of FSTEP(LR) with entry/removal thresholds.

Let me know if you'd like:

- The function to return the list of variables added/removed at each step
- A version that logs each step verbosely
- A plot of deviance vs steps or AIC

Want to add any other diagnostics or visualizations to this?
